# Metformin Beyond Glycemic Control: Cardiovascular Protection and Diabetes Prevention

**DOI:** 10.3390/jcdd13010033

**Published:** 2026-01-06

**Authors:** Georgios E. Zakynthinos, Georgios I. Tsironikos, Evangelos Oikonomou, Konstantinos Kalogeras, Gerasimos Siasos, Vasiliki Tsolaki

**Affiliations:** 1 3rd Department of Cardiology, “Sotiria” Chest Diseases Hospital, Medical School, National and Kapodistrian University of Athens, 11527 Athens, Greece; 2 Department of Medicine, University of Ioannina, University Campus, 45110 Ioannina, Greece; g.tsironikos@uoi.gr; 3 Cardiovascular Division, Brigham and Women’s Hospital, Harvard Medical School, Boston, MA 02115, USA; 4 Critical Care Department, University Hospital of Larissa, Faculty of Medicine, University of Thessaly, Mezourlo, 41335 Larissa, Greece; vasotsolaki@yahoo.com

**Keywords:** metformin, type 2 diabetes mellitus, cardiovascular disease, diabetes prevention, insulin resistance, glycemic control, heart failure, atherosclerosis, antidiabetic therapy

## Abstract

Metformin, the most widely prescribed oral antihyperglycemic agent, is established as the first-line therapy for type 2 diabetes mellitus (T2DM) owing to its efficacy, affordability, and safety. Increasing evidence indicates that its benefits extend beyond glycemic control, encompassing cardiovascular protection and diabetes prevention in individuals at elevated cardiometabolic risk. Mechanistic studies demonstrate that metformin exerts pleiotropic effects through activation of AMP-activated protein kinase, modulation of the gut microbiota, inhibition of pro-inflammatory and oxidative stress pathways, and improvements in endothelial function, lipid metabolism, and insulin sensitivity. These actions address core drivers of atherosclerosis and metabolic dysfunction, many of which occur independently of glucose lowering. In patients with T2DM, the cardiovascular benefits of metformin are well recognized, including reductions in all-cause mortality and cardiovascular events. In individuals without diabetes but at high cardiovascular risk—such as those with prediabetes, obesity, or metabolic syndrome—evidence is more limited, as most data are derived from subgroup analyses or trials with surrogate endpoints. Nonetheless, consistent signals suggest that metformin may delay the progression from prediabetes to overt diabetes and potentially confer vascular protection, particularly in carefully selected high-risk populations. Clinical trials and meta-analyses have demonstrated that metformin reduces incident diabetes by approximately one quarter in high-risk adults, with stronger effects observed in younger, overweight individuals, women with prior gestational diabetes, and those treated for longer durations. However, uncertainties remain regarding its long-term cost-effectiveness, optimal dosing strategies, and cardiovascular benefits in non-diabetic populations. The ongoing VA-IMPACT trial (NCT02915198) is expected to clarify whether metformin reduces major cardiovascular events in prediabetic patients with atherosclerotic disease. Taken together, metformin represents more than an antidiabetic drug. Its pleiotropic mechanisms, favorable safety profile, and low cost support its potential integration into broader cardiometabolic prevention strategies, including primary prevention. Expanding its role beyond diabetes management may offer a cost-effective, widely accessible intervention with significant public health impact.

## 1. Introduction

Cardiovascular disease (CVD) and type 2 diabetes mellitus (T2DM) are leading causes of global morbidity and mortality and exhibit a complex bidirectional relationship driven by shared pathophysiological mechanisms, including insulin resistance, chronic inflammation, endothelial dysfunction, and atherogenesis [1]. Their frequent coexistence amplifies individual cardiovascular risk, resulting in a disproportionately high burden of complications and increased healthcare costs [2,3]. This close interrelationship between T2DM and CVD highlights the urgent need for therapeutic strategies capable of addressing both conditions simultaneously [4].

Although multiple pharmacological options are available for glycemic control, concerns have been raised regarding their cardiovascular safety. Consequently, attention has increasingly focused on agents such as metformin, which may confer both glycemic and cardiovascular benefits.

Metformin, a biguanide antihyperglycemic agent, is among the most widely prescribed treatments for T2DM and offers substantial benefits for glucose regulation and diabetes-related complications. While the precise mechanisms underlying these effects remain incompletely understood, metformin continues to serve as the cornerstone of T2DM management due to its well-established efficacy, favorable safety profile—particularly its low risk of hypoglycemia and lactic acidosis in appropriately selected patients—and cost-effectiveness [5].

Contemporary clinical guidelines consistently endorse metformin as first-line therapy for T2DM. The American Diabetes Association (ADA) 2025 Standards of Care reaffirm metformin as the preferred initial pharmacologic treatment for patients without significant renal impairment. However, newer drug classes, including sodium–glucose cotransporter-2 (SGLT-2) inhibitors and glucagon-like peptide-1 (GLP-1) receptor agonists, are recommended for individuals at elevated cardiovascular or renal risk [6]. Similarly, the 2023 European Society of Cardiology (ESC) Guidelines recognize the metabolic and weight-related benefits of metformin, while prioritizing newer agents for patients with established cardiovascular disease [7].

Nonetheless, metformin’s prospective significance in cardiovascular protection continues to generate interest. In addition to earlier studies that showed metformin can lower the risk of complications and death from T2DM [8,9], a 2025 nationwide real-world retrospective cohort study found that metformin-treated patients with T2DM had a lower risk of acute myocardial infarction (AMI), showing that it can protect the heart in everyday clinical practice [10]. Additional observational data suggest that, in newly diagnosed T2DM patients, metformin administration—either as monotherapy or in conjunction with sulfonylureas—correlated with enduring decreases in cardiovascular (CV) events, overall mortality, and CV mortality over follow-up durations extending to a decade [11].

Due to the positive results in lowering CV events in people with T2DM, a number of RCTs have looked into metformin’s CV effects in people who do not have diabetes but have different levels of CVD risk. These earlier trials predominantly yielded negative or inconclusive outcomes, demonstrating no meaningful influence on carotid intima-media thickness (cIMT) or surrogate indicators of CVD in patients with coronary artery disease (CAD) undergoing statin therapy [12]. Similarly, no definitive cardiovascular advantage was identified in the general non-diabetic population, perhaps attributable to insufficient statistical power [13], nor in patients with coronary artery disease and a history of myocardial infarction without diabetes [14].

In the last ten years, more and more study has looked into metformin’s effects on the heart and metabolism as a whole. Consequently, metformin has surfaced as a viable preventative approach to inhibit the transition from prediabetes to T2DM, especially when combined with rigorous lifestyle modifications. The Diabetes Prevention Program (DPP) and its long-term follow-up (DPPOS) showed that metformin significantly slowed the progression from prediabetes to T2DM, especially in younger, overweight people and women who had gestational diabetes in the past [15,16]. Recent meta-analyses validate its efficacy in diabetes prevention within high-risk populations, indicating a substantial additive advantage when combined with lifestyle adjustments [17]. Despite this enduring preventive effect, a statistically significant reduction in major cardiovascular events was not observed over two decades, likely attributable to extensive statin and antihypertensive use, treatment arm crossover, and design-related factors [15,16]. In general, trials show that there is a difference between good surrogate markers and real-life cardiovascular outcomes.

The safety, tolerability, and cost-effectiveness of metformin further support its ongoing utilization. Long-term follow-up studies have demonstrated that gastrointestinal side effects are generally mild and transient, while modest weight loss is maintained over time [5,15]. Current guidelines stress the need for regular checks on kidney function, but they also confirm that metformin is quite safe based on decades of usage around the world [6,7,17].

In conclusion, metformin continues to be pivotal in the management of diabetes, the reduction in metabolic risk, and likely in the prevention of cardiovascular disease. Mechanistic and observational evidence clearly indicates cardioprotective advantages; yet, randomized studies in non-diabetic individuals remain unclear. Its capacity to postpone or avert T2DM in persons at elevated risk is well-documented; nonetheless, this has not resulted in conclusive enhancements in cardiovascular outcomes. Given the increasing global prevalence of cardiometabolic diseases and a developing treatment landscape that emphasizes cardiovascular and renal protection, a thorough reassessment of metformin’s present and prospective role is essential. This review combines mechanistic insights, epidemiological trends, and trial results to make its role in modern cardiometabolic care clearer.

## 2. Pharmacology and Mechanisms of Action of Metformin

Metformin has been in clinical use for more than six decades. Originally derived from *Galega officinalis* (French lilac), its glucose-lowering properties were first recognized in the 1920s; however, early guanidine derivatives were abandoned because of toxicity concerns and the concurrent availability of insulin. Interest in metformin re-emerged in the 1940s during antimalarial research, when clinical trials conducted for influenza unexpectedly demonstrated its antihyperglycemic effects. Subsequently, evidence accumulated in Europe supporting metformin’s ability to improve insulin resistance and manage adult-onset hyperglycemia without promoting weight gain or increasing the risk of hypoglycemia. Following extensive evaluation, metformin was ultimately approved by the U.S. Food and Drug Administration in 1995 [18].

### 2.1. Pharmacokinetics and Pharmacodynamics

Metformin has an oral bioavailability of about 55 ± 16%, and most of it is absorbed in the small intestine. It is not broken down by the body and is eliminated unchanged by the kidneys. In people with normal kidney function, it has a plasma half-life of around 5 h [19]. The drug does not bind to plasma proteins [20] and has a large volume of distribution (Vd/F) of 300–600 L at steady state with a daily dose of 2000 mg, which shows that it is taken up by tissues [19].

Transporters are mostly responsible for how metformin is absorbed and how it is used in the body. The plasma membrane monoamine transporter (PMAT) helps the intestines take in substances, whereas organic cation transporters (OCTs) control how the liver takes in substances and how the kidneys get rid of them. Genetic variations in these transporters may influence metformin pharmacokinetics and clinical outcomes, highlighting the significance of pharmacogenomics [19,21].

Pharmacodynamically, metformin diminishes hepatic glucose production, augments peripheral insulin sensitivity, and elevates glucose uptake by facilitating GLUT4 translocation in skeletal muscle and adipose tissue, without precipitating hypoglycemia [22].

### 2.2. AMPK Activation, Mitochondrial Effects and Inhibition of Gluconeogenesis

A central mechanism of action of metformin is the activation of AMP-activated protein kinase (AMPK), a key cellular energy sensor that regulates energy homeostasis. By inhibiting mitochondrial complex I, metformin reduces ATP production and increases intracellular AMP and ADP levels, leading to AMPK activation. This, in turn, suppresses the expression of gluconeogenic genes (e.g., PEPCK and G6Pase), decreases hepatic glucose production, enhances lipid oxidation, and increases glucose uptake in skeletal muscle, collectively improving insulin sensitivity and lowering fasting plasma glucose levels [22,23].

Beyond its mitochondrial effects, metformin can also activate AMPK through lysosomal signaling pathways, including the PEN2–ATP6AP1 complex and inhibition of SHIP2, highlighting mechanisms that are independent of changes in AMP/ADP ratios [24]. In addition, metformin suppresses hepatic gluconeogenesis by inhibiting mitochondrial glycerol-3-phosphate dehydrogenase (mGPD), thereby altering the hepatic redox state and limiting substrate availability for glucose production [19].

Notably, sodium–glucose cotransporter-2 (SGLT-2) inhibitors—initially developed for the management of type 2 diabetes—have also been shown to activate AMPK. This effect is thought to contribute to their pleiotropic benefits across cardiovascular, renal, pulmonary, digestive, and endocrine/metabolic systems through improvements in mitochondrial function, reductions in inflammation, and enhanced lipid oxidation. However, in contrast to SGLT-2 inhibitors, which are costly and often limited by access or tolerability, metformin achieves AMPK activation at a fraction of the cost. This shared mechanistic pathway underscores metformin’s enduring relevance as a low-cost, mechanistically robust agent in contemporary cardiometabolic therapy [25,26].

### 2.3. Gut Microbiome and Intestinal Mechanisms

New evidence suggests that the gastrointestinal system is a key part of how metformin works. Metformin alters the gut microbiota by elevating Akkermansia muciniphila and Lactobacillus, while diminishing Bacteroides. These modifications enhance glucose metabolism and inflammatory regulation [27]. These changes boost the production of short-chain fatty acids (SCFAs), make the intestinal barrier work better, and increase the release of GLP-1, which makes insulin work better and helps control blood sugar levels [28].

Metformin builds up in enterocytes at levels that are up to 300 times greater than those in plasma. This speeds up anaerobic glycolysis and the formation of lactate. The resultant lactate engages in hepatic gluconeogenesis within a fruitless, energy-depleting cycle [28,29]. Evidence from animal models further underscores the gut’s significance: mice deficient in intestinal AMPKα1 exhibit a diminished glucose-lowering response to metformin, suggesting the involvement of gut–endocrine pathways and potentially brown adipose tissue thermogenesis [30].

Recent research by Shi et al. using high-fat diet (HFD) mice indicated that metformin augmented beneficial bacteria, including Lactobacillus, and elevated short- and medium-chain fatty acids, specifically butyric, valeric, and lauric acids. These alterations improved HFD-induced disruptions in glucose and lipid metabolism and diminished liver injury, corroborating the notion that metformin’s metabolic advantages are partially facilitated by the manipulation of the gut microbiota [31].

### 2.4. Anti-Inflammatory and Antioxidant Effects

Metformin’s anti-inflammatory properties are partially mediated through AMPK activation, which alters immune cell metabolism from glycolysis to oxidative phosphorylation, consequently diminishing NF-κB signaling—a key regulator of innate immunity—and restricting cytokine release [24,32]. Metformin increases antioxidant defenses and reduces reactive oxygen species (ROS) via acting on mitochondria and activating AMPK, thereby protecting cells from metabolic stress [21,32].

### 2.5. Lipid Modulation and Weight Effects

Metformin has demonstrated efficacy in lowering triglycerides, LDL cholesterol, and total cholesterol, presumably through AMPK-mediated inhibition of lipogenic enzymes, including ACC1 and HMG-CoA reductase, the rate-limiting enzyme in cholesterol production [33]. In the liver, these activities inhibit lipogenesis and promote fatty acid oxidation, leading to enhanced lipid profiles and decreased triglyceride/cholesterol production. Recent pharmacodynamic studies have further substantiated these benefits, demonstrating beneficial control of lipid metabolism through transporter and AMPK pathways [21].

Moluski et al. found that metformin-treated mouse and human primary hepatocytes exhibited enhanced expression of the sterol transporters ABCG5/8 (ATP-binding cassette transporters G5/G8) and the bile salt export pump (Bsep) through AMPK activation. These results lend partial support to the hypothesis that metformin may confer cardiovascular benefits by enhancing reverse cholesterol transport, while also indicating the upregulation of low-density lipoprotein receptor (LDLr) expression, potentially increasing hepatic cholesterol uptake [34]. These lipid alterations, albeit less significant than those induced by statins, may synergistically contribute to a reduction in cardiovascular risk.

Moreover, metformin is weight-neutral or slightly weight-reducing, partially due to elevated levels of growth differentiation factor-15 (GDF15), which diminishes hunger and food consumption. Gut–brain and intestinal-BAT signaling loops support this [30].

In addition, clinical and experimental evidence suggests that metformin modestly increases endogenous glucagon-like peptide-1 (GLP-1) levels, which may contribute to its weight-reducing effects. GLP-1 promotes satiety, delays gastric emptying, and suppresses appetite through central hypothalamic pathways and gut–brain signaling. Although the increase in endogenous GLP-1 induced by metformin is modest compared with the pharmacological activation of the GLP-1 receptor achieved by GLP-1 receptor agonists, studies in both diabetic and non-diabetic individuals have demonstrated that metformin enhances GLP-1 secretion following nutrient intake, likely through increased intestinal L-cell secretion [35,36,37]. This incretin-based mechanism may act synergistically with growth differentiation factor-15 (GDF15) to promote mild but clinically meaningful weight loss.

In summary, these mechanisms make metformin a drug that works in many ways, including through metabolic, vascular, inflammatory, and possibly microbiome-mediated pathways. Its capacity to affect several pathogenic processes highlights its therapeutic promise in the management of cardiometabolic diseases.

## 3. Evidence of Cardiovascular Protection of Metformin in Patients with Type 2 Diabetes

The cardiovascular effects of metformin in patients with type 2 diabetes mellitus (T2DM) have been investigated for more than three decades, beginning with the landmark UK Prospective Diabetes Study (UKPDS). In UKPDS 34, 753 overweight patients with newly diagnosed T2DM were randomized to intensive glycemic control with metformin or to conventional therapy, which primarily consisted of dietary management. Over a median follow-up of 10.7 years, metformin treatment was associated with significant reductions in the risk of any diabetes-related endpoint (32%, *p* = 0.002), diabetes-related mortality (42%, *p* = 0.017), and all-cause mortality (36%, *p* = 0.011) [38].

In a secondary analysis, outcomes in 342 overweight patients allocated to metformin were compared with those of 951 overweight patients receiving intensive glucose control with chlorpropamide (n = 265), glibenclamide (n = 277), or insulin (n = 409). Metformin demonstrated superior clinical outcomes compared with these agents, including lower rates of any diabetes-related endpoint (*p* = 0.0034), all-cause mortality (*p* = 0.021), and stroke (*p* = 0.032). A combined analysis of the main and supplementary cohorts further confirmed fewer diabetes-related outcomes among metformin-treated patients, corresponding to a 19% relative risk reduction (95% CI, 2–33; *p* = 0.033) [38].

Extended post-trial follow-up over an additional 10 years compared patients initially assigned to conventional therapy (dietary restriction) with those allocated to intensive therapy (sulfonylurea, insulin, or metformin in overweight patients). This analysis demonstrated a sustained “legacy effect” of metformin, characterized by persistent cardiovascular protection, including ongoing reductions in microvascular complications and emerging decreases in myocardial infarction and all-cause mortality, despite comparable glycemic control across treatment groups. These findings suggest that early initiation of metformin may confer durable cardioprotective benefits, particularly in overweight patients with T2DM [39]. A summary of clinical studies that have supported—or failed to clearly demonstrate—cardiovascular benefits of metformin in patients with T2DM is provided in Table 1.

In 2013, Hong and colleagues conducted a multicenter randomized clinical trial in China comparing metformin with glipizide in 304 patients with type 2 diabetes mellitus (T2DM) and pre-existing coronary artery disease (CAD), including prior myocardial infarction (MI) or ≥50% stenosis of a major coronary artery. Participants were randomized to receive either glipizide (30 mg daily) or metformin (1.5 g daily) for three years. Over a median follow-up of 5.0 years, the composite major adverse cardiovascular events (MACE) endpoint—including cardiovascular death, all-cause death, nonfatal MI, nonfatal stroke, or arterial revascularization—was significantly reduced in the metformin group (hazard ratio [HR] 0.54; 95% CI 0.30–0.90; *p* = 0.026), providing early evidence of cardiovascular benefit beyond the UKPDS era [40] (see Table 1 for clinical studies supporting the cardiovascular benefit of metformin in patients with T2DM).

However, the cardiovascular benefits of metformin have been questioned in both earlier and more recent trials and meta-analyses. For example, Lamanna et al. did not find a clear association between metformin use and reduced cardiovascular risk, particularly when combined with sulfonylureas (SUs) [41]. Similarly, a large network meta-analysis by Palmer et al., which included 301 clinical trials evaluating nine classes of glucose-lowering drugs, either alone or in combination, found no significant differences in cardiovascular or all-cause mortality between drug classes. The authors concluded that metformin, whether as monotherapy or in combination, had a neutral-to-beneficial effect on MACE. However, many of the included trials were as short as 24 weeks, which may have been insufficient to detect meaningful differences in cardiovascular outcomes [42].

Around the same time, Griffin et al. performed a meta-analysis of 13 randomized controlled trials comprising 2079 patients with T2DM, most of whom were overweight or obese and younger than 65 years. This study assessed various doses and formulations of oral metformin against placebo, lifestyle intervention, or no intervention, evaluating both mortality and cardiovascular outcomes. The UKPDS contributed the majority of data, accounting for 52.3% of the weight for MI and 70.5% for stroke. While all outcomes except stroke favored metformin, none reached statistical significance, leaving uncertainty regarding metformin’s efficacy in reducing cardiovascular risk in T2DM, despite its status as the recommended first-line therapy [43].

In alignment with these results, Gnesin et al., in a review of 18 RCTs, also did not demonstrate a significant correlation between metformin monotherapy (as opposed to no intervention or alternative glucose-lowering agents) and a decreased risk of all-cause mortality or cardiovascular events in patients with T2DM [48]. Xu et al. performed a comparable meta-analysis assessing metformin’s impact on cardiovascular outcomes, including major adverse cardiovascular events (MACE), hospitalization for heart failure, stroke, and acute myocardial infarction (MI), and identified no substantial benefit over non-metformin therapies based on observational studies. Additionally, metformin did not demonstrate superiority over DPP-4 inhibitors in mitigating MACE risk [44] (See Table 1 for clinical studies that failed to clearly demonstrate cardiovascular benefit).

Even though these outcomes are not clear, there is a lot of other evidence that metformin protects the heart. By 2019, Han et al. aggregated data from 40 studies encompassing over 1,060,000 CAD patients, illustrating that metformin use correlated with markedly reduced adjusted hazard ratios for cardiovascular mortality (aHR 0.81), all-cause mortality (aHR 0.67), and cardiovascular events (aHR 0.83), with supplementary advantages noted in patients with a history of myocardial infarction and heart failure. Metformin was also more effective than SUs in reducing cardiovascular events [14].

In 2020, Petrie et al. provided a critical reappraisal of metformin’s cardiorenal outcomes, reaffirming its foundational role in T2DM management based on UKPDS findings, while cautioning against extrapolating results from older RCT designs to the standards of contemporary CV outcomes trials [45]. In the same year, Zhang et al. released a meta-analysis that showed that treating T2DM with metformin lowered the risk of cardiovascular events, including death and new cases. The authors, however, recognized significant variation among research, which may constrain the robustness of the conclusions [8].

The Italian Society of Diabetology and the Italian Association of Clinical Diabetologists created new rules for the pharmaceutical treatment of T2DM in 2021. As part of this approach, Monami et al. did a meta-analysis of RCTs that lasted at least 52 weeks and compared metformin to placebo, no therapy, or active comparators. They looked at how oral anti-diabetic medications (OADs) affected MACE and death from all causes. Metformin was substantially correlated with a reduced risk of MACE. The decrease in all-cause mortality did not achieve statistical significance overall; however, it attained significance upon the exclusion of RCTs comparing metformin with SUs, SGLT-2 inhibitors, or GLP-1 receptor agonists. The authors determined that these results indicate a slight yet constant advantage of metformin, with a propensity to decrease all-cause mortality in comparison to placebo or alternative oral antidiabetic drugs [46].

At the same time, Italian longitudinal retrospective cohort research utilizing the Catalan SIDIAP database (Information System for the Development of Research in Primary Care) evaluated newly diagnosed T2DM patients commencing first-line monotherapies. Metformin diminished the risk of all evaluated outcomes, including MACE, MI, stroke, all-cause mortality, heart failure (HF), and peripheral artery disease (PAD). Conversely, individuals administered dipeptidyl peptidase-4 inhibitors (DPP-4i) or repaglinide exhibited elevated risks of major adverse cardiovascular events (MACE) and mortality in comparison to those treated with metformin [47].

In 2022, Schernthaner et al. examined mechanistic and clinical outcome data, emphasizing metformin’s cardiovascular advantages through mechanisms such as AMPK activation, anti-inflammatory effects, and endothelial protection—mechanisms that remain pertinent in the context of contemporary outcome trials [54].

A 2023 study by Li compiled previous advancements and empirical evidence, indicating that metformin use correlated with a 24% decrease in all-cause mortality and a 32% decrease in cardiovascular mortality, including reduced incidences of coronary artery disease and heart failure among several patient groups with cardiovascular disease. Nonetheless, no significant impact was observed on the incidence of myocardial infarction, angina pectoris, or stroke [49]. In a retrospective population-based cohort study of T2DM patients treated with either SUs or metformin mono-therapy, Zhou et al. found that metformin significantly reduced the risk of stroke, cardiovascular disease mortality, and all-cause mortality compared to SUs, while also significantly lowering the risk of atrial fibrillation [50]. An umbrella review corroborated that metformin use diminished the risk of CVD events, all-cause mortality, and CVD mortality by 17%, 20%, and 23%, respectively [51].

Bahardoust et al. [11] performed a pooled analysis of three prospective cohort studies (TLGS, MESA, ARIC) examining the effects of metformin and sulfonylureas on cardiovascular and mortality outcomes relative to diabetes duration in newly diagnosed T2DM patients by 2025. People who were already using other OADs such SGLT-2 inhibitors or insulin, or who already had CVD, were not included. In a cohort of 4108 T2DM patients, each additional year of metformin therapy, whether administered alone or in conjunction with sulfonylureas, resulted with a 7% reduction in the hazard of cardiovascular events, a 4% decrease in all-cause mortality, and a 6% decline in cardiovascular mortality. Combined metformin-sulfonylurea therapy resulted in an approximate 10% reduction in major cardiovascular events and a 12% decrease in cardiovascular disease mortality, maintained for up to 10 years [11].

Recent statewide real-world retrospective cohort research assessed the impact of metformin on the prevention of acute myocardial infarction in patients with type 2 diabetes mellitus. The trial comprised 9186 metformin users and an equivalent number of matched non-users, monitored over a span of 7 years. MI was the main result. The utilization of metformin was correlated with a markedly reduced risk of myocardial infarction (adjusted hazard ratio 0.76; 95% confidence interval 0.41–0.96), and this protective impact was uniform across both genders. The scientists concluded that metformin may have a dual function, facilitating glycemic control and mitigating cardiovascular risk [10].

In the same year, Chen et al. conducted a retrospective analysis of 8675 postoperative cardiac surgery patients admitted to intensive care units, revealing that postoperative metformin use was correlated with decreased short- and long-term all-cause mortality, thereby affirming its efficacy even in acute cardiovascular stress scenarios [52].

It is crucial to compare metformin with newer glucose-lowering medications that are now indicated in guidelines for their cardiovascular beneficial properties [6,7]. In this context, Wong et al. assessed the lifetime advantages of metformin compared to FDA-approved SGLT-2 inhibitors in T2DM patients with pre-existing CVD. Both treatments lowered the risk of death from all causes and heart disease compared to placebo. But patients who took SGLT2 inhibitors were more likely to die from any cause (HR 1.308, 95% CI 1.103–1.550) than those who took metformin. The estimated survival gains free from all-cause mortality were 23.26 months with metformin and 23.04 months for SGLT-2 inhibitors. These results indicate that metformin has significant cardiovascular advantages that are at least on par with those of SGLT-2 inhibitors [53].

The progression of evidence on metformin’s cardiovascular effects in T2DM has transitioned from an initial phase of enthusiasm to ambiguous findings, and more recently, to increasingly consistent indications of benefit. The 2013 RCT by Hong et al. offered preliminary controlled evidence indicating that metformin surpassed sulfonylureas (glipizide) in mitigating major adverse cardiovascular events (MACE), however constrained by sample size and patient selection [40]. Han et al.’s 2019 meta-analysis of over one million patients showed big drops in CV and all-cause mortality. However, because it was based on observational data, it was hard to draw causal conclusions [14].

Later studies confirmed similar results. Petrie et al. underscored the deficiencies of earlier trial designs in relation to contemporary cardiovascular outcomes trials [46]. Smaller meta-analyses and reviews centered on RCTs contributed supportive, but inconclusive, findings [8,47]. Mechanistic investigations conducted in 2022 delineated biologically feasible pathways—encompassing AMPK activation, anti-inflammatory effects, and endothelial impacts—that corroborate the reported benefits [48]. Li’s 2023 synthesis indicated significant decreases in overall and cardiovascular mortality [49]. Bahardoust et al. (2025) demonstrated duration-dependent advantages, indicating that prolonged metformin usage reduces the risks of cardiovascular events and mortality over a ten-year period [11].

Recent studies indicate that the advantages of metformin may be analogous to those of SGLT-2 inhibitors and GLP-1 receptor agonists [53]. Further empirical evidence, including a national survey indicating a decreased incidence of acute myocardial infarction [10] and ICU data reflecting enhanced outcomes post-cardiac surgery [52], reinforces its cardioprotective function.

Overall, while early trials were inconclusive, accumulating evidence from large cohort studies, mechanistic insights, and long-term analyses increasingly indicates that metformin confers cardiovascular protection beyond glucose lowering, particularly with sustained use in high-risk T2DM populations. Unanswered questions include the best time to take it, how to combine it with newer drugs, and the best dose and length of time to take it.

## 4. Cardiovascular Outcomes in Patients with Elevated Cardiovascular Risk but Without Diabetes: Is There Evidence to Add Metformin?

Metformin’s cardioprotective potential has also been explored in populations without diabetes but with elevated cardiovascular risk, including individuals with metabolic syndrome, insulin resistance, or prediabetes. Many of the pathophysiological processes that drive atherosclerosis—such as low-grade inflammation, endothelial dysfunction, dyslipidemia, and insulin resistance—are already present well before the clinical onset of type 2 diabetes. However, the evidence in these populations remains more limited than in patients with diabetes, although small clinical trials and mechanistic studies provide insights into metformin’s potential utility (see Table 2 for a summary of clinical studies that, respectively, support or fail to demonstrate cardiovascular protection by metformin in non-diabetic individuals at elevated cardiovascular risk).

An early randomized, double-blind trial in 33 non-diabetic women with angina and angiographically normal coronary arteries (placebo: n = 17; metformin: n = 16) evaluated metformin 500 mg twice daily for eight weeks. Treatment improved endothelial microvascular function, Duke treadmill score, and angina frequency, suggesting both vascular and symptomatic benefit, although interpretation was limited by the small sample size and short duration [55] (see Table 2 for clinical studies supporting cardiovascular benefit of metformin in non-diabetic patients).

In 2019, the multicenter CODYCE trial assessed metformin in 258 propensity score–matched patients with stable angina and nonobstructive coronary stenosis, including both normoglycemic and prediabetic individuals. Compared with untreated prediabetic patients, metformin use improved coronary endothelial function, measured by acetylcholine-induced vasodilation, and reduced the incidence of major adverse cardiovascular events (MACE) over 24 months, indicating lower cardiovascular risk through improved endothelial function [56]. Around the same period, a study in patients undergoing coronary artery bypass grafting (CABG) evaluated pericoronary fat inflammation and the leptin-to-adiponectin ratio. Prediabetic patients treated with metformin for approximately six months prior to surgery experienced fewer MACE and exhibited lower pericoronary inflammatory tone than non-users, supporting an anti-inflammatory mechanism of cardiovascular benefit [57].

More recently, in 2023, Zheng et al. applied systems biology and Mendelian randomization approaches in 338,425 non-diabetic participants from the UK Biobank. Genetic variants affecting seven metformin-related targets were linked to favorable changes in body mass index (BMI), systolic and diastolic blood pressure, and, theoretically, reduced risk of coronary artery disease [58].

In their comprehensive review, Schernthaner et al., while noting that although the pleiotropic actions provide a strong biological rationale for cardioprotection, most supportive data still derive from diabetic populations, with limited and underpowered evidence in high-risk non-diabetic cohorts [54].

Experimental and clinical studies suggest that metformin may exert CV benefits extending beyond glucose lowering, particularly in HF and endothelial dysfunction. In HF with preserved Ejection Fraction (HFpEF)-like mouse models, metformin reduced myocardial stiffness, improved diastolic function, and preserved exercise capacity by enhancing titin compliance [59]. Clinical evidence echoes these findings: a systematic review and meta-regression by Halabi et al. reported reduced mortality in HF patients, with greater benefit in HFpEF (EF > 50%) [60]. Similarly, a 2022 meta-analysis of nine RCTs found regression of left ventricular mass index and modest improvements in ejection fraction, particularly at doses > 1000 mg/day and in patients with established HF [61]. These findings highlight a potential role for metformin in improving both systolic and diastolic function, though they primarily relate to HF rather than coronary artery disease.

Luo et al. argued that these data point toward a paradigm shift, with metformin potentially conferring CV protection even in individuals without diabetes, through mechanisms involving inflammation, lipid metabolism, and other pleiotropic effects. They emphasized its cost-effectiveness and accessibility as an adjunct for both primary and secondary prevention [62].

Supporting this view, a JACC State-of-the-Art Review on angina with nonobstructive coronary arteries emphasized the importance of specialized coronary function testing and a systematic approach that integrates lifestyle, pharmacological, and device-based therapies to improve outcomes while noting that metformin may have beneficial effects in this context [63], an observation based largely on the small but suggestive trial by Jadhav et al. [55].

However, the evidence supporting metformin’s benefits in prediabetic patients at high CV risk remains limited. Despite favorable signals from selected studies, most observational and real-world data indicate that metformin does not provide significant cardiovascular protection in this population (See Table 2 for clinical studies that do not support cardiovascular benefit of metformin in non-diabetic patients at elevated cardiovascular risk).

The CAMERA (Carotid Atherosclerosis: Metformin for Insulin Resistance) trial was a randomized, double-blind, placebo-controlled study of 173 non-diabetic individuals with coronary heart disease and central obesity, all receiving statin therapy. Over 18 months, participants were randomized to metformin or placebo, with cIMT progression as the primary endpoint. No significant differences were observed in cIMT or carotid plaque score. Although metformin reduced weight and waist circumference, these changes did not translate into anti-atherosclerotic effects. The authors concluded that in this high-risk, statin-treated, non-diabetic population, metformin offered no additional CV benefit [12].

The Diabetes Prevention Program (DPP) and its follow-up study (DPPOS) evaluated whether metformin or intensive lifestyle intervention could reduce cardiovascular events in individuals with impaired glucose tolerance. Participants were randomized to metformin, lifestyle modification, or placebo for three years, after which all groups received a less intensive lifestyle program, while metformin therapy was continued in the original metformin arm. Over 21 years of follow-up, both interventions significantly reduced progression to type 2 diabetes but did not decrease rates of major cardiovascular events, including stroke, myocardial infarction, or cardiovascular death. Adjustment for risk factors did not change these results, likely due to treatment crossover, widespread use of statins and antihypertensives, declining adherence, and off-study metformin use [16].

Additionally, a large meta-analysis by Han et al., which evaluated outcomes according to diabetes status, found that metformin use was associated with significant reductions in all-cause mortality, cardiovascular mortality, and cardiovascular events in patients with T2DM. In contrast, no significant benefit was observed in non-diabetic patients with coronary artery disease or a history of myocardial infarction, suggesting that the cardiovascular protective effects of metformin may be largely confined to populations with diabetes [16].

Therefore, despite encouraging signals in selected studies and several plausible mechanisms-such as anti-inflammatory effects and favorable metabolic actions-the majority of real-world data suggests little or no CV benefit of metformin in non-diabetic or prediabetic individuals. Dihoum et al. [64] note that while mechanistic rationale exists, most clinical trials in non-diabetic populations have been small, underpowered, and conducted before the widespread use of SGLT-2 inhibitors and GLP-1 receptor agonists, leaving clinical benefit inconclusive. Similar to SGLT-2 inhibitors, metformin activates AMPK—a key energy-sensing enzyme implicated in cardioprotection—yet does so through distinct pathways, at significantly lower cost [25,26]. However, current evidence does not support the routine use of metformin for CV risk reduction in non-diabetic patients, even those at elevated risk.

Nevertheless, the potential of metformin to improve CV outcomes in high-risk, non-diabetic populations remains an open question. The ongoing VA-IMPACT trial (Investigation of Metformin in Pre-Diabetes on Atherosclerotic Cardiovascular Outcomes, NCT02915198) is a large, multicenter, randomized, double-blind, placebo-controlled study testing whether metformin can reduce mortality and major adverse CV events in adults with prediabetes and established atherosclerotic CV disease. Approximately 7410 participants are being randomized to metformin (up to 2000 mg/day) or placebo, with all receiving lifestyle counseling. Initiated in April 2023 and expected to conclude in March 2029, this trial is anticipated to provide critical evidence on the long-term CV effects of metformin in this population.

## 5. Prevention of Type 2 Diabetes in At-Risk Populations

Preventing the development of type 2 diabetes mellitus (T2DM) in high-risk populations is a major public health priority, given the growing global burden of diabetes and its complications. Prediabetes is defined by impaired fasting glucose (IFG), impaired glucose tolerance (IGT), or elevated hemoglobin A1c (HbA1c) levels between 5.7% and 6.4% [65]. Additional factors that further increase risk include a history of gestational diabetes, polycystic ovary syndrome, a family history of diabetes in first-degree relatives, age over 45 years, and membership in higher-risk ethnic groups, such as Hispanic/Latino, Native American, African American, Asian American, Alaska Native, or Pacific Islander populations [65]. Lifestyle and metabolic contributors include poor diet [66], physical inactivity (fewer than three exercise sessions per week), overweight or obesity, and central adiposity [67]. Other associated conditions include non-alcoholic fatty liver disease, hypertension or use of antihypertensive therapy, hypertriglyceridemia (>250 mg/dL) and/or low HDL cholesterol (<35 mg/dL), cardiovascular disease, acanthosis nigricans, and HIV infection [65].

Metformin has been extensively studied for delaying or preventing progression from prediabetes to overt T2DM, particularly in individuals with IFG, IGT, or metabolic syndrome (see Table 3 for a summary of clinical trials evaluating the role of metformin in preventing type 2 diabetes in at-risk populations). A landmark multicenter randomized clinical trial enrolled 3234 individuals with IFG and IGT, who were randomized to lifestyle intervention, metformin (850 mg twice daily), or placebo [68]. Over 2.8 years, metformin reduced diabetes incidence by 31% compared with placebo, whereas lifestyle intervention reduced it by 58% (both *p* < 0.001). The number needed to treat to prevent one case of diabetes over three years was 6.9 for lifestyle intervention and 13.9 for metformin. While both approaches effectively reduced diabetes incidence, lifestyle intervention was more efficacious. Subgroup analyses indicated that metformin was particularly effective in individuals under 60 years of age, those with a body mass index (BMI) ≥ 35 kg/m^2^, and women with a history of gestational diabetes. These findings provide strong evidence supporting targeted metformin use in high-risk populations [68].

Over 15 years of follow-up in the DPP and DPPOS studies, diabetes incidence was reduced by 27% with lifestyle intervention and by 18% with metformin compared with placebo, although the differences between groups diminished over time. At year 15, the cumulative incidence of diabetes was 55% with lifestyle intervention, 56% with metformin, and 62% with placebo [69]. These findings were reinforced by the DPPOS, which extended follow-up to 21 years [16].

Meanwhile, a 2008 meta-analysis confirmed that metformin significantly reduced the risk of progression to T2DM in high-risk individuals [70].

Herman [71], using data from the landmark DPP/DPPOS Research Group, evaluated the interventions’ resource use, medical costs, effects on diabetes progression, quality of life, and overall cost-effectiveness. Although lifestyle and metformin interventions were initially more expe nsive than placebo, these costs were offset by reduced medical expenditures in the non-intervention groups. Quality of life was consistently higher with lifestyle intervention, and both the 10-year trial and lifetime simulation analyses confirmed that lifestyle and metformin were highly cost-effective, and in some cases cost-saving, compared with placebo [71].

A recent 2024 meta-analysis of 12 randomized controlled trials including 2720 participants compared lifestyle plus metformin versus lifestyle alone. The combination significantly reduced the incidence of T2DM and lowered HbA1c, with fasting plasma glucose also declining at 12 months. However, changes at 3 and 6 months were comparable between groups, and no significant differences were observed in secondary outcomes such as blood pressure, plasma lipids, or weight [72].

Similarly, a 2023 review and meta-analysis confirmed metformin’s effectiveness in high-risk individuals, particularly those with impaired glucose regulation. Metformin significantly reduced the incidence of T2DM, with the greatest benefit observed in younger adults, individuals with higher BMI, and women with a history of gestational diabetes, reinforcing the findings of the DPP/DPPOS Research Group. Its efficacy, however, appeared diminished in individuals aged 60 years or older. These results underscore metformin’s role in diabetes prevention but also highlight its persistent underuse, even among high-risk groups, reflecting a gap between evidence and practice [73].

Reflecting this implementation gap, a 2024 retrospective primary care study examined metformin use in 372 high-risk prediabetic patients, as defined by the American Diabetes Association. Although nearly 85% met ADA criteria for metformin, only 60% had been prescribed the drug. Prescriptions were more common in patients with higher BMI, elevated glucose, and a documented diagnosis of prediabetes [74].

An updated meta-analysis published in mid-2025 reassessed the overall effectiveness of metformin in preventing T2DM using the most recent evidence [17]. Metformin reduced the incidence of T2DM by 23% in high-risk adults and by 25% in individuals with prediabetes (*p* < 0.0001). Subgroup analyses indicated greater benefit among prediabetic participants, predominantly female cohorts, and individuals over 60 years of age. Protective effects were observed in both overweight/obese and normal-weight groups, with the strongest results seen at a daily dose of 1700 mg and treatment durations of at least 18 months; benefit diminished after discontinuation. Combination with lifestyle interventions further amplified effectiveness, reducing diabetes incidence in prediabetic patients by 52% compared with standard care, with some benefit even at lower metformin doses (e.g., 500 mg/day). The analysis also suggested that Caucasian populations derived the greatest benefit.

Despite these findings, concerns remain regarding consistency across trials and meta-analyses. While the preventive effect of metformin in high-risk and prediabetic individuals is well established-and certain associations are robust, such as dose, duration, and loss of benefit after discontinuation-differences persist. For example, the landmark DPP trial by Knowler et al. found that metformin was most effective in those under 60 years of age and with BMI ≥ 35 kg/m^2^ [68], conclusions echoed in a subsequent meta-analysis that relied heavily on this study [73]. By contrast, the more recent meta-analysis by Tsironikos et al. [17], which included 13 RCTs-many newly published-with 5329 participants (2603 metformin; 2726 controls), reported stronger benefit in individuals over 60 years of age and across BMI categories. Notably, this recent study demonstrated low heterogeneity, enhancing confidence in its findings.

Limitations of the studies, particularly in meta-analyses, stem largely from differences in the primary populations examined, depending on the main risk factor under investigation. For most RCTs, prediabetes is the sole or the main risk factor [68,75,76,77,78,79,80,81,82,83,84]. In other studies, the main risk factor is overweight/obesity [68,79,83,85], CVD [83,86,87,88], central adiposity [12,83,85], HIV infection [75,77] and family history of T2DM in first-degree relatives [79].

The number of T2DM risk factors included also varies between studies, ranging from one [76,78,80,82,86,87], two [12,68,75,77,85], to multiple factors [79,83].

Closely related to these limitations, T2DM is assessed either as the primary outcome [68,75,76,77,80,82,87], or as the secondary outcome [12,79,86]. When secondary, the primary outcomes often includes: changes in body weight and in metabolic and lipidemic profile in patients with overweight/obesity and central adiposity [85], metformin sensitivity and glucose tolerance in patients with prediabetes [79], the progression of mean distal carotid intima–media thickness in patients with CAD [12], CV risk profile in patients with CAD [86], and left ventricular hypertrophy in patients with CAD and prediabetes [88].

Further variability exists in intervention characteristics. Study duration ranges from several months to years, and the total daily metformin dose varies from 500 mg to 2000 mg, while comparator arms also differs, including placebo, lifestyle modification, other glucose-lowering therapies, or combinations thereof [76,77,79,81].

## 6. Metformin and Ischemia–Reperfusion Injury: Indications for Cardiac and Cerebral Protection

### 6.1. Cardiac Ischemia–Reperfusion Injury

A substantial body of experimental and translational evidence demonstrates that metformin confers significant protection against myocardial ischemia–reperfusion (I/R) injury. Acute or short-term administration before or at the onset of reperfusion reduces infarct size, preserves left ventricular systolic function, and enhances myocardial ATP recovery. Early experimental studies showed that metformin augments I/R-induced AMPK activation, increases endothelial nitric oxide synthase (eNOS) phosphorylation, and limits oxidative injury-mechanisms that occur independently of glycemic effects [89]. Similarly, metformin through transient AMPK activation during early reperfusion has been shown to reduce infarct size by nearly 35% in rat models; infarct size was reduced from 47.8 +/− 1.7% to 31.4 +/− 2.9% [90].

More recent experimental investigations confirm these findings using advanced molecular and imaging techniques. Metformin has been shown to preserve mitochondrial homeostasis after I/R, reduce cardiomyocyte apoptosis, and modulate autophagy–mitophagy balance, as demonstrated by TSPO-targeted PET imaging [91]. Additional work indicates that metformin mitigates reperfusion injury through inhibition of PANoptosis and modulation of the c-Jun N-terminal kinase (JNK) pathway [92], stabilizes Hypoxia Inducible Factor-1α (HIF-1α) signaling under chronic hypoxia protecting the heart [93] and prevents early reperfusion-related mitochondrial permeability transition pore opening [94]. Collectively, these studies support a robust, AMPK-centered cardioprotective mechanism at clinically achievable doses.

### 6.2. Cerebral Ischemia–Reperfusion Injury

Metformin also exerts protective effects in cerebral ischemia–reperfusion (I/R) injury. In models of transient global ischemia, metformin pretreatment activates AMPK, upregulates antioxidant pathways (notably increased the level of Nuclear factor erythroid 2-related factor (Nrf2) and heme oxygenase-1 (HO-1) in the hippocampus of ischemic rats), enhances the level of glutathione and catalase activities and suppresses neuroinflammatory mediators such as TNF-α, NF-κB, and COX-2 [95]. In addition, enhances neuronal survival, reduces infarct volume, through AMPK-dependent pathways [96].

Recent studies further support its relevance in stroke: combined sinomenine–metformin therapy reduces cerebral I/R damage through suppression of NLRP3 inflammasome activation and enhancement of PINK1/Parkin-mediated mitophagy in diabetic stroke [97]. Metformin also exerts neuroprotective effects by reducing neuronal apoptosis and modulating the JNK/p38 MAPK signaling pathways in middle cerebral artery occlusion models [98] and protects against hyperglycemia-aggravated cerebral I/R injury via regulating AMPK/ULK1/PINK1/Parkin-dependent mitophagy and apoptosis [99]. Additional work reveals its protective effects on astrocytic calcium signaling in during mild hypoxia [100].

To our knowledge, a first-in-human, ongoing trial is currently evaluating the safety, feasibility, and pharmacokinetics of metformin in full-term infants with hypoxic–ischemic encephalopathy (perinatal brain injury), aiming to explore its potential neurorestorative effects in a high-risk population. Using physiologically based pharmacokinetic modeling, the study employs a dose-escalation protocol to inform future trials and marks an important step in translating preclinical neuroprotective findings into clinical practice [101].

The evidence from myocardial and cerebral I/R models demonstrates that metformin engages core cellular stress-response pathways-particularly sustained AMPK activation, mitochondrial stabilization, improved nitric oxide signaling, attenuation of oxidative stress, and autophagy/mitophagy regulation. However, since most of these findings derive from preclinical studies in rodent models, large-scale outcome trials in humans are urgently needed to confirm the translational relevance and therapeutic potential of metformin in ischemic injury.

## 7. Metformin and Polycystic Ovary Syndrome (PCOS)

Metformin has long been integral to the management of polycystic ovary syndrome (PCOS), a complex endocrine–metabolic disorder characterized by insulin resistance, compensatory hyperinsulinemia, and hyperandrogenism; notably, women with PCOS often exhibit multiple cardiovascular disease (CVD) risk factors, including dyslipidemia, hypertension, central obesity, and endothelial dysfunction [102]. By improving hepatic and peripheral insulin sensitivity, metformin lowers circulating insulin levels, thereby reducing ovarian androgen production and restoring menstrual cyclicity and ovulatory function in many women with PCOS. Moreover, metformin improves the impairments typically observed in hyperinsulinemic PCOS patients, reducing the possible evolution towards metabolic syndrome and Type 2 diabetes; and when pregnancy occurs, it consistently reduces the risk of gestational diabetes, eclampsia and hypertension [103,104]. These mechanisms are further supported by the AMPK signaling pathway, which is essential for maintaining reproductive homeostasis and plays a key role in regulating steroidogenesis, implantation, and embryonic development. Dysregulation of AMPK signaling has been implicated in several female reproductive disorders, including PCOS [105]. Notably, metformin enhances granulosa cell function by reducing TNF-α- and chemokine-mediated inflammation through an AMPK-dependent mechanism [106]. Clinical studies have also demonstrated that metformin improves reproductive outcomes, including increased pregnancy and live birth rates. A large randomized trial reported significantly higher pregnancy rates with metformin compared to placebo (53.6% vs. 40.4%), particularly among obese women (49.0% vs. 31.4%), as well as higher live birth rates (41.9% vs. 28.8%) [107]. It may also improve endothelial function and arterial stiffness, indicating broader cardiovascular benefit in this high-risk population [102]. Importantly, its benefits are not limited to overweight women: in normal-BMI PCOS patients, metformin improved menstrual frequency and androgen levels comparably to chiglitazar [108]. Furthermore, a recent trial comparing empagliflozin to metformin in overweight women with PCOS and prediabetes suggested greater metabolic benefit with the SGLT2 inhibitor, although adjusted differences were not statistically significant [109].

## 8. Safety and Practical Considerations of Metformin

Metformin is generally well tolerated. The most common adverse effects are gastrointestinal, including nausea, abdominal discomfort, bloating, and diarrhea. These symptoms are dose-dependent and typically transient, often resolving within the first weeks of therapy, and can be reduced by taking the drug with meals. Because metformin is eliminated renally, adequate kidney function is required. Current guidelines recommend avoiding initiation if estimated glomerular filtration rate (eGFR) is <45 mL/min/1.73 m^2^ and discontinuing treatment if eGFR falls below 30 mL/min/1.73 m^2^. Recent evidence indicates that metformin may still be used with caution in patients with moderate renal impairment, provided renal function is closely monitored [110].

The most serious, though extremely rare, adverse event is metformin-associated lactic acidosis (MALA). Because metformin is excreted unchanged by the kidney, MALA most often occurs in patients with renal impairment or in those with risk factors for acute kidney injury. Mechanistically, metformin can raise plasma lactate levels in a concentration-dependent manner through inhibition of mitochondrial respiration, usually in the presence of renal dysfunction or secondary conditions impairing lactate clearance [19,111,112].

## 9. Conclusions

Metformin has progressed from a primary treatment for T2DM to a versatile medication with increasing evidence for extensive cardiometabolic advantages. In addition to glycemic management, it may aid in the prevention of cardiovascular disease and postpone the onset of diabetes in persons at high risk who do not yet have diabetes.

Mechanistic and observational studies indicate that these effects are facilitated by many pathways, including AMPK activation, modification of the gut microbiota, prevention of inflammatory cascades, and enhancements in endothelial function, lipid metabolism, and insulin sensitivity. These pathways collectively address the primary factors contributing to atherosclerosis and metabolic dysfunction, with several seemingly operating independently of glucose reduction, highlighting metformin’s potential as more than merely an antidiabetic agent.

The cardiovascular advantages of metformin are well established in persons with type 2 diabetes mellitus (T2DM). Conversely, evidence in non-diabetic adults at increased cardiovascular risk is scarce, as the majority of data is derived from subgroup analyses or trials employing surrogate endpoints instead of conclusive cardiovascular outcome trials. Nonetheless, its mechanistic plausibility, acceptable safety profile, and affordability recommend cautious application in meticulously chosen high-risk patients, especially those with prediabetes, obesity, or metabolic syndrome. Stratification methods that identify such individuals—for instance, by BMI, components of metabolic syndrome, or a history of gestational diabetes—may enhance patient selection.

There are still some important limits. Unresolved inquiries include long-term cost-effectiveness, optimal dosage regimens, and regulatory concerns associated with off-label usage in cardiometabolic prevention. It is essential to conduct large-scale cardiovascular outcome trials in non-diabetic groups. Results from the ongoing VA-IMPACT trial (NCT02915198), expected in 2029, are likely to provide critical evidence.

Even with these questions, metformin’s many effects, safety, and low cost make it a good choice for cardiometabolic preventive regimens, including primary prevention. Adding it to larger cardiovascular and metabolic frameworks could be a cost-effective and broadly available intervention with important effects on public health.

Despite the emergence of SGLT2 inhibitors and GLP-1 receptor agonists—both proven to confer substantial cardiovascular and renal benefits in diverse populations—metformin remains clinically and mechanistically relevant. While newer agents derive their cardioprotective credibility from large contemporary outcome trials, metformin’s benefits are supported by landmark studies, long-term follow-up data, and a strong biological rationale rooted in AMPK activation, anti-inflammatory pathways, and improved metabolic flexibility. Although it may not achieve the same magnitude or consistency of MACE reduction, metformin’s affordability, long-standing safety, and weight-neutral profile support its ongoing role as a foundational therapy. In this evolving therapeutic landscape, metformin complements rather than competes with newer agents, offering a widely accessible tool for cardiometabolic risk reduction.

## Figures and Tables

**Table 1:** A: Clinical Studies Supporting Cardiovascular Benefit of Metformin in patients with Type 2 Diabetes. B: Clinical Studies Failing to Clearly Demonstrate Cardiovascular Benefit of Metformin in patients with Type 2 Diabetes.

Study/Year	Population/Study Design	Intervention	Main CV Findings	Interpretation/Limitations
UKPDS 34, 1998 [38]	Open-label randomized trial; 753 overweight patients with newly diagnosed T2DM	Metformin vs. conventional therapy (diet); Median 10.7 years	Metformin reduced any diabetes-related endpoint by 32%, diabetes-related death by 42%, and all-cause mortality by 36%	Landmark trial; overweight, newly diagnosed T2DM; older standards of care
UKPDS 34 secondary analysis, 1998 [38]	Overweight patients; 342 metformin vs. 951 SU/insulin (chlorpropamide, (n = 265), glibenclamide (n = 277) or insulin (n = 409)	Metformin vs. SU/insulin; Median 10.7 years	Metformin showed lower rates of any diabetes-related endpoint, all-cause mortality, and stroke; Combined analysis: 19% RRR in diabetes-related outcomes	Post hoc secondary comparison; background therapies heterogeneous
Holman et al., 2008 [39]	Overweight patients from original UKPDS cohorts	Conventional (diet) vs. intensive therapy (including metformin) 10-year monitoring post-trial	Sustained “legacy effect”: persistent reduction in microvascular complications, MI and all-cause mortality in metformin group despite convergent HbA1c	Observational follow-up; treatments after trial not fully controlled
Hong et al., 2013 [40]	Multicenter RCT; 304 patients with CAD (prior MI or ≥50% stenosis)	Metformin 1.5 g/day vs. SU (glipizide 30 mg/day); treatment 3 years; median follow-up 5 years	Expanded MACE (CV death, all-cause death, MI, stroke, revascularization) reduced with metformin	Moderate sample size; but strong signal vs. SU
Han et al., 2019 [14]	Meta-analysis of 40 studies (>1,060,000 CAD patients	Metformin users vs. non-users/other OADs	Metformin associated with lower CV mortality, all-cause mortality, and CV events	Treatment selection bias
Zhang et al. 2020 [8]	Meta-analysis of T2DM cohorts	Metformin vs. non-metformin	Reduced CV risk (mortality and event incidence) with metformin	Substantial heterogeneity; potential publication bias
Bahardoust et al., 2025 [11]	Pooled analysis of 3 prospective cohorts (4108 patients); newly diagnosed T2DM, no pre-existing CVD; excluded SGLT-2i/insulin	Metformin (±SU) therapy	Each additional year of metformin therapy reduced hazards of CV events by 7%, all-cause mortality by 4%, CV mortality by 6%. Metformin+SU combination: 10% reduction in major CV events and 12% reduction in CVD mortality over up to 10 years	Treatment duration not randomized; possible adherence bias
Chang et al., 2025 [10]	Nationwide retrospective cohort; 9186 metformin users vs. 9186 matched non-users	Metformin vs. non-metformin	Over 7 years, metformin associated with lower risk of acute MI, consistent in both sexes	Matching study, but retrospective; outcome restricted to MI
Lamanna et al., 2011 [41]	Meta-analysis of RCTs; focus on metformin ± SUs	Metformin vs. non-metformin, including SU combinations	No clear association between metformin and reduced CVD risk (especially when combined with SUs)	Many older, small RCTs; heterogeneous comparators; limited CV-specific endpoints
Palmer et al., 2016 [42]	Network meta-analysis of 301 trials, 9 drug classes, various combinations	Comparisons across metformin, SUs, DPP-4i, TZDs, insulin, …	No significant differences in CV or all-cause mortality between drug classes; metformin monotherapy or in combination appeared neutral-to-beneficial for MACE	Many trials as short as 24 weeks, which likely is too brief to detect CV differences
Griffin et al., 2017 [43]	Meta-analysis: 13 RCTs, 2079 adults (mainly ≤65 years, overweight/obese)	Metformin vs. placebo, lifestyle, or no intervention	All outcomes except stroke numerically favored metformin, but none not reached statistical significance	RCT pool small; dominated by UKPDS study
Xu et al., 2022 [44]	Meta-analysis of observational studies	Metformin vs. non-metformin therapy; include comparison with DPP-4i	No difference in metformin for MACE (HF hospitalization, stroke, MI); no superiority vs. DPP-4i for MACE	Confounding by indication; heterogeneity across cohorts and endpoints
Petrie et al., 2020 [45]	Critical narrative review of cardiorenal outcomes	Metformin vs. non-metformin	Reaffirmed metformin’s foundational role based on UKPDS; highlighted need for cautious interpretation vs. SGLT-2i/GLP-1 receptor agonist data	Mainly RCTs prior to modern CVOT era
Monami et al., 2021 [46]	Meta-analysis of RCTs ≥ 52 weeks; basis for Italian Society of Diabetology recommendations	Metformin vs. placebo, no treatment, or active comparators	Metformin significantly reduced MACE, but not all-cause mortality	Exclusion choices influence effect estimates
Italian SIDIAP cohort, 2021 [47]	Observational longitudinal real-world cohort; newly diagnosed T2DM	First-line metformin vs. DPP-4i, repaglinide, others	Metformin reduced risk of MACE, and all-cause death	Observational; prescribing patterns and comorbidities differ by drug
Gnesin et al., 2020 [48]	Systematic review: 18 RCTs	Metformin monotherapy vs. no intervention or other agents	No significant association between metformin monotherapy and reduced all-cause mortality or CVD events.	Limited event numbers and duration
Li et al., 2023 [49]	Synthesis/pooled analysis and real-world data	Metformin users vs. non-users	Metformin associated with 24% reduction in all-cause mortality and 32% reduction in CV mortality	Heterogeneity across included studies; varying endpoint definitions
Zhou et al., 2022 [50]	Retrospective cohort	Metformin vs. SU monotherapy	Metformin significantly reduced risk of stroke, CVD mortality, all-cause mortality, and atrial fibrillation compared with SUs	Retrospective observational design; possible confounding by patient selection and comorbidities
Bahardoust et al., 2024 [51]	Umbrella review of multiple meta-analyses	Metformin vs. non-metformin	Metformin use reduced CVD events, all-cause mortality, and CVD mortality by 17%, 20%, and 23%, respectively	Quality of underlying meta-analyses varies; many based on non-randomized data
Chen et al., 2025 [52]	Retrospective ICU cohort; 8675 postoperative cardiac surgery patients	Postoperative metformin use vs. no metformin	Postoperative metformin use associated with reduced short- and long-term all-cause mortality	Perioperative management may differ between groups
Wong et al., 2024 [53]	Modeling/comparative effectiveness; T2DM with established CVD	Metformin vs. SGLT-2 inhibitors vs. placebo	Both metformin and SGLT-2i reduced all-cause and CV mortality vs. placebo. SGLT-2i group had higher all-cause mortality risk vs. metformin	

**Table 2:** Cardiovascular Outcomes in Patients with Elevated Cardiovascular Risk but Without Diabetes: Is There Evidence to Add Metformin? A. Evidence Supporting Cardiovascular Protection By Metformin in Non-Diabetic Patients With Elevated Cardiovascular Risk. B. Evidence Not Supporting Cardiovascular Protection By Metformin in Non-Diabetic Patients With Elevated Cardiovascular Risk.

Study/Year	Population	Study Design/Intervention	Main CV Findings	Interpretation/Limitations
Jadhav et al., 2006 [55]	33 non-diabetic women with angina and normal coronaries	Randomized, double-blind trial; metformin 500 mg BID vs. placebo for 8 weeks	Improved endothelial microvascular function, Duke treadmill score, and angina frequency	Promising vascular improvement, but very small and short-duration study
Sardu et al., 2019 [56]	258 normoglycemic or prediabetic patients with stable angina and non-obstructive CAD	Propensity-matched study; metformin vs. no metformin for 24 months	Improved coronary endothelial function and lower MACE	Suggests endothelial benefit
Sardu et al., 2019 [57]	Prediabetic patients undergoing CABG	Observational; ~6-month pre-surgery metformin use vs. non-use	Lower pericoronary inflammatory tone and fewer MACE in metformin users	Supports anti-inflammatory cardioprotection; not randomized study
Zheng et al., 2023 [58]	338,425 non-diabetic UK Biobank participants	Mendelian randomization and systems biology	Lower BMI, blood pressure, and theoretical lower CAD risk	Mechanistic genomic evidence, but not proof of clinical benefit
Schernthaner et al., 2022 [54]	High-risk non-diabetic populations	Mechanistic clinical review	AMPK activation, anti-inflammatory and endothelial pathways support potential CV benefit	Strong biological rationale; no clinical evidence
Slater et al., 2019 [59]	HFpEF phenotype mice	Experimental study	Metformin improved exercise capacity; improved myocardial diastolic function	HF benefit independent of glycemia
Halabi et al., 2020 [60]	HF patients	Systematic review and meta-regression	Reduced HF mortality with metformin, particularly in HFpEF	Suggests benefit in HF; relevance to CAD uncertain
Kamel et al., 2022 [61]	HF patients	Meta-analysis of 9 RCTs	Regression of LV mass; modest improvement in EF at higher metformin doses	Indicates structural cardiac benefit; primarily HF-focused
Luo et al., 2019 [62]	Cardiometabolic risk patients	Review	Highlights anti-inflammatory, lipid-modifying, endothelial mechanisms	Hypothesis-generating
Smilowitz et al., 2023 [63]	Angina with nonobstructive coronary arteries (ANOCA)	Expert clinical review	Notes potential benefit of metformin in microvascular angina	Based largely on a small trial; evidence still preliminary
Preiss et al., 2014 [12]	173 non-diabetic adults with CAD and central obesity on statins (CAMERA)	Randomized, double-blind, placebo-controlled trial (18 months)	No improvement in cIMT or carotid plaque score; reduced body weight but no anti-atherosclerotic effect	High-quality negative evidence against metformin’s CV benefit in non-diabetics
Goldberg et al., 2022 [16]	Prediabetic adults with impaired glucose tolerance (DPP/DPPOS)	RCT (3 years) vs. lifestyle vs. placebo and 21-year follow-up	Metformin reduced progression to diabetes but did not reduce MACE	Strong evidence against CV benefit in prediabetes
Han et al., 2019 [14]	CAD patients with and without diabetes	Meta-analysis	CV benefit seen in diabetics only; no significant benefit in non-diabetics	Suggests metformin’s cardioprotection may be diabetes-dependent
Dihoum et al., 2023 [64]	Non-diabetic high-risk individuals	Clinical review	No evidence to recommend metformin	Lack of robust clinical benefit; but existing trials are underpowered

HFpEF, Heart failure with preserved ejection fraction; RCT, Randomized control trial; MACE, Major Adverse Cardiovascular Events; cIMT, Carotid Intima-Media Thickness; DPP, Diabetes Prevention Program; DPPOS, Diabetes Prevention Program Outcomes Study.

**Table 3:** Prevention of Type 2 Diabetes in At-Risk Populations.

Study/Year	Population	Study Design/Intervention	Key Findings on Diabetes Prevention	Interpretation
Knowler et al., 2002 [68]	3234 adults with IFG and IGT (DPP trial)	RCT; metformin 850 mg BID vs. lifestyle vs. placebo over 2.8 years	Metformin reduced diabetes incidence by 31% and lifestyle by 58% vs. placebo; Strongest metformin effect in age < 60, BMI ≥ 35, and women with GDM history	Landmark evidence supporting targeted metformin use for diabetes prevention.
DPP/DPPOS, 2015 [69]	DPP participants	Long-term follow-up to 15 years	Diabetes incidence reduced by 27% with lifestyle, 18% with metformin vs. placebo; differences narrowed with time	Long-term benefit persists but attenuates; treatment crossover and changing therapies dilute effect size
DPPOS, 2022 [16]	DPP participants	Extended observational follow-up 21 years	No significant reduction in major CV events despite diabetes prevention	Metformin effective for diabetes prevention but does not reduce CV events in prediabetes
Salpeter et al., 2008 [70]	High-risk individuals (prediabetes/IFG/IGT)	Meta-analysis	Metformin significantly reduced progression to T2DM vs. control	Metformin effective for diabetes prevention
Herman 2015 [71]	DPP/DPPOS cohort	Cost-effectiveness analysis	Metformin and lifestyle highly cost-effective; reduced long-term medical expenditures; lifestyle improves quality of life	Supports metformin as cost-effective public health intervention in high-risk populations
Amer et al., 2024 [72]	12 RCTs, 2720 participants High-risk adults; lifestyle ± metformin	Meta-analysis Metformin + lifestyle vs. lifestyle alone	Combination therapy reduced diabetes incidence, HbA1c and fasting glucose at 12 months	Combination approach superior for glycemic outcomes; benefits appear time-dependent
Patel et al., 2023 [73]	High-risk individuals (IFG/IGT/GDM history)	Systematic review and meta-analysis	Metformin effective for preventing T2DM; strongest effects in younger adults, higher BMI, and GDM history; reduced efficacy ≥ 60 years	Metformin effective for diabetes prevention; highlights persistent underuse in eligible patients
Alvarez-Villalobos et al., 2024 [74]	372 high-risk adults meeting ADA criteria	Retrospective primary care observational study	Only 60% of ADA-eligible patients were prescribed metformin; more likely in patients with higher BMI, glucose, or explicit prediabetes diagnosis	Demonstrates underutilization of metformin in real-world practice despite guidelines
Tsironikos et al., 2025 [17]	13 RCTs; 5329 high-risk adults	Meta-analysis Metformin vs. control	Metformin reduce T2DM incidence by 23% overall and 25% in prediabetes. Strongest effect with ≥18 months therapy and dose of 1700 mg/day. Benefit persisted across age and BMI groups	Largest and most updated review; shows broader and more consistent benefit, even in adults > 60 years. Indicates dose- and duration-dependent effectiveness

IFG, Impaired Fasting Glucose; IGT, Impaired Glucose Tolerance.

## Data Availability

No new data were created or analyzed in this study. Data sharing is not applicable to this article.

## Abbreviations

The following abbreviations are used in this manuscript:

ACC1	Acetyl-CoA Carboxylase 1
ADA	American Diabetes Association
aHR	Adjusted Hazard Ratio
AMI	Acute Myocardial Infarction
AMPK	AMP-Activated Protein Kinase
ATP	Adenosine Triphosphate
BMI	Body Mass Index
Bsep	Bile Salt Export Pump
CABG	Coronary Artery Bypass Grafting
CAD	Coronary Artery Disease
CAMERA	Carotid Atherosclerosis: Metformin for Insulin Resistance
CI	Confidence Interval
CODYCE	Coronary Artery Disease in Young Adults with Coronary Endothelial Dysfunction Trial
CV	Cardiovascular
CVD	Cardiovascular Disease
cIMT	Carotid Intima-Media Thickness
DPP	Diabetes Prevention Program
DPPOS	Diabetes Prevention Program Outcomes Study
DPP-4i	Dipeptidyl Peptidase-4 Inhibitors
EF	Ejection Fraction
ESC	European Society of Cardiology
eGFR	Estimated Glomerular Filtration Rate
FDA	Food and Drug Administration
GDF15	Growth Differentiation Factor-15
GLP-1	Glucagon-Like Peptide-1
HbA1c	Hemoglobin A1c
HF	Heart Failure
HFpEF	Heart Failure with Preserved Ejection Fraction
HIV	Human Immunodeficiency Virus
HMG-CoA	3-Hydroxy-3-Methylglutaryl-CoA
HR	Hazard Ratio
ICU	Intensive Care Unit
IFG	Impaired Fasting Glucose
IGT	Impaired Glucose Tolerance
LDL	Low-Density Lipoprotein
LDLr	Low-Density Lipoprotein Receptor
MACE	Major Adverse Cardiovascular Events
MALA	Metformin-Associated Lactic Acidosis
MI	Myocardial Infarction
NF-κB	Nuclear Factor Kappa-Light-Chain-Enhancer of Activated B Cells
OADs	Oral Antidiabetic Drugs
PAD	Peripheral Artery Disease
RCT	Randomized Controlled Trial
ROS	Reactive Oxygen Species
SCFAs	Short-Chain Fatty Acids
SGLT-2	Sodium–Glucose Cotransporter-2
SUs	Sulfonylureas
T2DM	Type 2 Diabetes Mellitus
TLGS	Tehran Lipid and Glucose Study
UKPDS	UK Prospective Diabetes Study
VA-IMPACT	Investigation of Metformin in Pre-Diabetes on Atherosclerotic Cardiovascular Outcomes
